# Three strategies to stabilise nearly monodispersed silver nanoparticles in aqueous solution

**DOI:** 10.1186/1556-276X-7-151

**Published:** 2012-02-22

**Authors:** Amadeus PZ Stevenson, Duani Blanco Bea, Sergi Civit, Sonia Antoranz Contera, Alberto Iglesias Cerveto, Sonia Trigueros

**Affiliations:** 1 Department of Physics, University of Oxford, Parks Road, Oxford, OX1 3PU, UK; 2 Department of Materials, National Centre for Scientific Research, PO Box 6414, Avenida 25 and 158, Cubanacán, Playa, Havana, CP 12100, Cuba; 3 Department of Statistics, University of Barcelona, Avenida Diagonal 645, Barcelona, 08028, Spain; 4 Institute of Nanoscience for Medicine, Oxford Martin School, 34 Broad Street, University of Oxford, Oxford, OX1 3BD, UK

**Keywords:** silver nanoparticles, stability, functionalisation, monodispersed, aging, toxicity.

## Abstract

Silver nanoparticles are extensively used due to their chemical and physical properties and promising applications in areas such as medicine and electronics. Controlled synthesis of silver nanoparticles remains a major challenge due to the difficulty in producing long-term stable particles of the same size and shape in aqueous solution. To address this problem, we examine three strategies to stabilise aqueous solutions of 15 nm citrate-reduced silver nanoparticles using organic polymeric capping, bimetallic core-shell and bimetallic alloying. Our results show that these strategies drastically improve nanoparticle stability by distinct mechanisms. Additionally, we report a new role of polymer functionalisation in preventing further uncontrolled nanoparticle growth. For bimetallic nanoparticles, we attribute the presence of a higher valence metal on the surface of the nanoparticle as one of the key factors for improving their long-term stability. Stable silver-based nanoparticles, free of organic solvents, will have great potential for accelerating further environmental and nanotoxicity studies.

**PACS: **81.07.-b; 81.16.Be; 82.70.Dd.

## Background

Metal nanoparticles have generated great interest for applications in physics, materials, chemistry and biomedical sciences in plasmonics [1], biosensing [2-4], nanomedicine [5-7], nanoelectronics [8], catalysis [9,10], magnetic fluids [11] and dye-based solar cells [12] due to their chemical, electronic, optical and magnetic properties. These applications depend on the availability of homogeneous nanoparticles of controlled size and shape, which remain stable in their complex target environments [13,14]. For example, metal nanoparticles exhibit surface plasmon resonance in the visible spectrum range, resulting in light scattering and characteristic absorbance peaks whose location and width depend on the type of metal, size and shape of the nanostructure and the medium they are immersed in [15-19]. Interactions between nanoparticles and biological matter will also depend on their size, shape and surface charge as they interact with different organisms [20].

Silver and gold nanoparticles have attracted great interest for many applications due to their strong plasmonic properties and to the availability of methods for synthesis [21,22]. The colloidal method has been extensively used due to the ability to synthesise nanoparticles directly in aqueous solution [23]. However, controlling the size and shape of metal nanoparticles remains challenging; nanoparticles are often heterogeneous in size and shape unless multiple reaction parameters are carefully regulated [22,24]. Synthesis via multiple steps, seed-mediated growth or via organic solvents has overcome several of these problems, although these synthesis methods increase in complexity with the number of steps involved and will limit potential biomedical applications when organic solvents are used [25-27].

The long-term stability of nanoparticles critically depends on the medium they are immersed in. The liquid influences interparticle forces and chemical reactivity, which affect aggregation, size and shape of the nanoparticles and long-term stability ('aging'), especially if the nanoparticles are applied or stored in aqueous conditions [28]. These effects are particularly relevant to more reactive metals such as silver. Stable silver nanoparticles in solution are necessary to apply and assess their interactions with biological matter and living cells. It is difficult to determine the effect of silver nanoparticle solutions where variable sizes, aggregates, surfactants and especially free silver ions are present [29].

A critical step to control the stability of inorganic nanoparticles lies in their surface modification and functionalisation [30,31]. Here, we compare three strategies to functionalise nearly monodispersed silver-based nanoparticles directly in aqueous solution. We have synthesised silver nanoparticles with a narrow shape and size distribution (15 nm quasi-spherical nanoparticles), functionalising them with polyethylene glycol (PEG) [32-34], gold (core-shell) and chromium (alloy). We have characterised the different silver-based nanostructures and established their stability by UV-Visible (UV-Vis) spectroscopy, transmission electron microscopy (TEM) and amplitude modulation atomic force microscopy (AFM) in liquid. We have tested the validity of our results using statistical bootstrapping tools, demonstrating a close relationship between homogeneity of nanoparticle size, shape and particle sphericity. Finally, this study has made it possible to produce stable silver-based nanoparticles that can be used for further applications and studies of toxicity and environmental impact, where the effect of the nanoparticle-containing solution can be attributed to nanoparticles of a controlled size and stability and not to by-products of synthesis (free ions, toxic surfactants), aggregation and/or degradation due to aging effects.

## Materials and methods

Unless otherwise stated, all reagents were purchased from Sigma-Aldrich (Dorset, UK), of analytical grade, and were used as received. Milli-Q water (Millipore Co., Billerica, MA, USA; specific resistivity of 18 MΩ cm) was used for the preparation of all solutions. Glassware was cleaned with Milli-Q water prior to the synthesis processes. Muscovite mica was purchased from SPI Supplies (West Chester, PA, USA).

### Nanoparticle synthesis

Silver (Ag) nanoparticles capped with citrate were obtained using the Turkevich method [35]. A solution of silver nitrate (125 ml, 1 mM) was heated to 95°C, and a solution of trisodium citrate (7.75 mM) was added. Nearly 15 min later, a colour change was observed indicating the formation of nanoparticles, and the solution was cooled to room temperature and stored at 5°C. During synthesis, we maintained a constant pH of 6.5.

PEG-functionalised silver nanoparticles (AgPEG) were obtained via direct PEGylation [32] by immediately adding a solution of PEG-6000 (80 mM) to freshly synthesised (as mentioned) silver nanoparticles (50 ml). The sample was cooled to -5°C for 8 h before storing at 5°C.

Silver-gold (Ag-Au) nanoshells were obtained by the successive reduction of two metal salts. A fresh solution of silver nanoparticles was prepared as previously described, then immediately heated to 90°C, and a solution of hydrogen tetrachloroaurate (1.2 mM) was added. The reaction mixture was then heated to 95°C, and at this point, a solution of trisodium citrate (7.75 mM) was added. When a colour change was observed, the mixture was cooled gradually to room temperature and stored at 5°C. During the second reaction, chloride ions produced from the reduction of hydrogen tetrachloroaurate reacted with unbound silver forming a silver halide which sedimented rapidly and was removed from the product.

Silver-chromium (AgCr) alloy nanoparticles were synthesised by co-reducing two metal salts. A solution of potassium dichromate dissolved in sulphuric acid (5 ml, 42 mM) was diluted twice in water (10 ml) and added to a solution of silver nitrate (125 ml, 1 mM) before heating to 95°C. A solution of trisodium citrate (7.75 mM) was then added. When a colour change was observed, the mixture was cooled to room temperature and stored at 5°C.

### Nanoparticle characterisation

UV-Vis absorption spectra of nanoparticle aqueous solutions were obtained using an MDR-23 monochromator (LOMO, St. Petersburg, Russia). TEM images were obtained using a Tecnai 12 TEM (FEI Company, Eindhoven, The Netherlands) and recorded with a Gatan US1000 2 K CCD camera (Gatan, Inc., Pleasanton, CA, USA). All samples were deposited on formvar-coated 200 mesh copper grids, and the excess liquid wicked off with filter paper. The samples were air-dried and examined unstained. TEM images were analysed in ImageJ 1.43 u (NIH, Bethesda, MD, USA). The area and perimeter of each nanoparticle was extracted before calculating circularity $\left(4\pi \left(\frac{\text{Area}}{\text{Perimete}{\text{r}}^{\text{2}}}\right)\right)$ and diameter $\left(2\sqrt{\frac{\text{Area}}{\pi }}\right)$, estimating particles to be spherical. Both metrics were sensitive to pixel density, with smaller particles yielding larger rounding errors due to the lower number of pixels available.

For atomic force microscopy imaging in liquid (Ag, AgCr), nanoparticle samples (2 μl) were diluted in NaCl buffer (48 μl, 20 mM with 50 mM HEPES, pH 6.5) and bath sonicated (Ultrawave U300, Ultrawave, Cardiff, UK) for 15 min. Samples were incubated on freshly cleaved mica for 20 min at room temperature before imaging in NaCl buffer. Due to PEG hydration, the height of AgPEG nanoparticles characterised by AFM in liquid (data not shown) was found to be twice the value of the diameter from TEM. Consequently, AgPEG nanoparticles were characterised in air by adding 2 μl of nanoparticle sample onto freshly cleaved mica and air-dried before imaging. Ag-Au nanoparticles were imaged in air after an incubation time of 2 h on freshly cleaved mica coated with poly-L-lysine (0.01%). Samples were imaged with a commercial MFP-3D AFM (Asylum Research, Santa Barbara, CA, USA) operating in amplitude modulation mode. Olympus AC240 (*k *= 2 N m^-1^) silicon cantilever tips in air and Olympus TR800/400 (*k *= 0.57 N m^-1^/0.08 N m^-1^) (all from Olympus Europa Holding GmbH, Hamburg, Germany) silicon nitride cantilevers in liquid were used. Low scan rates (0.3 Hz) further minimised tip sample forces from displacing deposited nanoparticles. Post-scan processing included image flattening and particle size analysis using the manufacturer's provided software (MFP-3D 080501 + 1429), based in Igor Pro 6.04 (WaveMetrics, Inc., Portland, OR, USA). Nanoparticle heights were calculated by thresholding height in topography images and extracting the *z *range of each particle.

To quantify the relationship between homogeneity of nanoparticle size, shape and aspect ratio, TEM diameters and AFM heights of nanoparticles were combined and described according to the eccentricity statistic distribution *ε *(Equation 1) via non-parametric bootstrapping:

(1) $$\varepsilon =\sqrt{\frac{a^{2}-b^{2}}{a^{2}}}=\sqrt{1-{\left(\frac{b}{a}\right)}^{2}}\;0<\varepsilon <1,$$

where *a *and *b *are half of the ellipse's major and minor axes (diameter and height), respectively. As the eccentricity value ranges from 0 to 1, the corresponding nanoparticle shape will range from spherical (*ε *= 0) to ellipsoidal (*ε *= 1). The sphericity of nanoparticles that resulted from different stabilisation strategies were then compared using the Kruskal-Wallis hypothesis test [extensive details are provided in Additional file 1].

## Results and discussion

### Silver nanoparticles functionalised with PEG: organic stability

**Figure 1:**
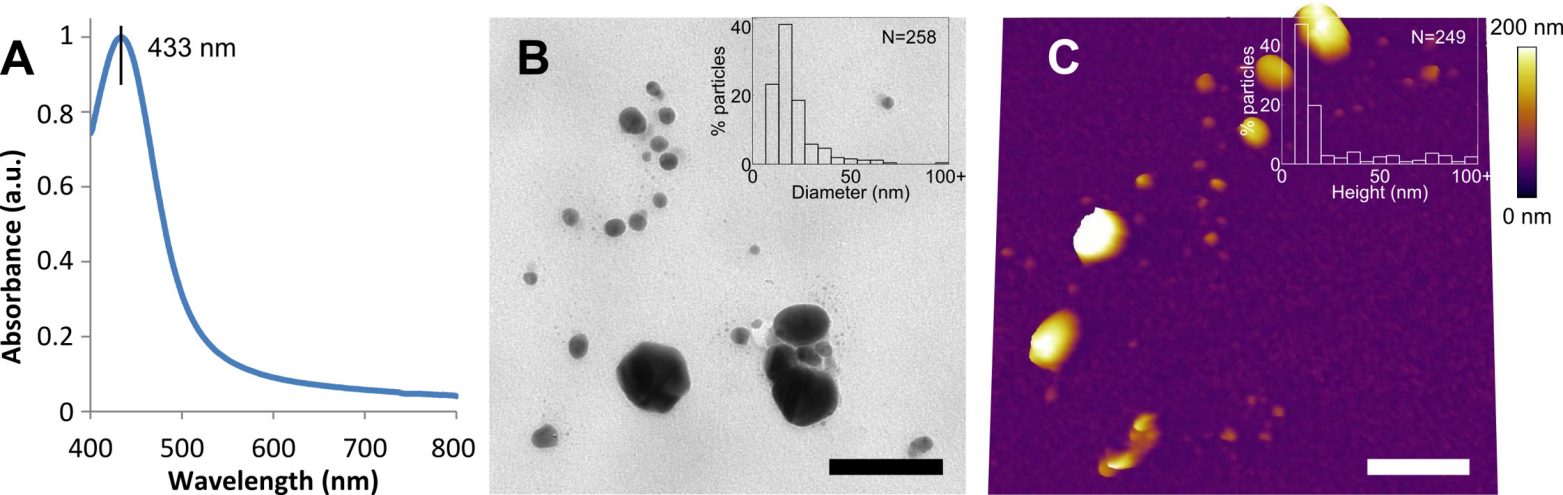
**Characterisation of silver nanoparticles**. (**A**) UV-Vis absorbance (arbitrary units) spectrum showing narrow absorption peak at 433 nm. (**B**) TEM image showing heterogeneous nanoparticles 100 days post-synthesis (scale bar 100 nm), with inset particle diameter distribution. (**C**) AFM topography image in liquid showing heterogeneous nanoparticles 100 days post-synthesis (scale bar 700 nm), with inset particle height distribution.

The synthesised citrate-capped silver nanoparticles were characterised by UV-Vis spectroscopy, TEM and AFM (Figure 1). The UV-Vis spectrum of the nanoparticles exhibited a narrow absorption peak at 433 nm due to plasmon resonance (Figure 1A), indicating a narrow size and shape distribution immediately post-synthesis [36]. TEM and AFM data obtained 100 days after synthesis gave a median particle diameter and height of about 14 nm (Figure 1B) and 10 nm (Figure 1C), respectively. Both TEM and AFM data show strong heterogeneity in the size and shape of synthesised nanoparticles. Large (> 100 nm in height) aggregates were observed directly in liquid (Figure 1C), confirming that the instability of the observed silver nanoparticles was not due to drying-induced aggregation during TEM sample preparation.

**Figure 2:**
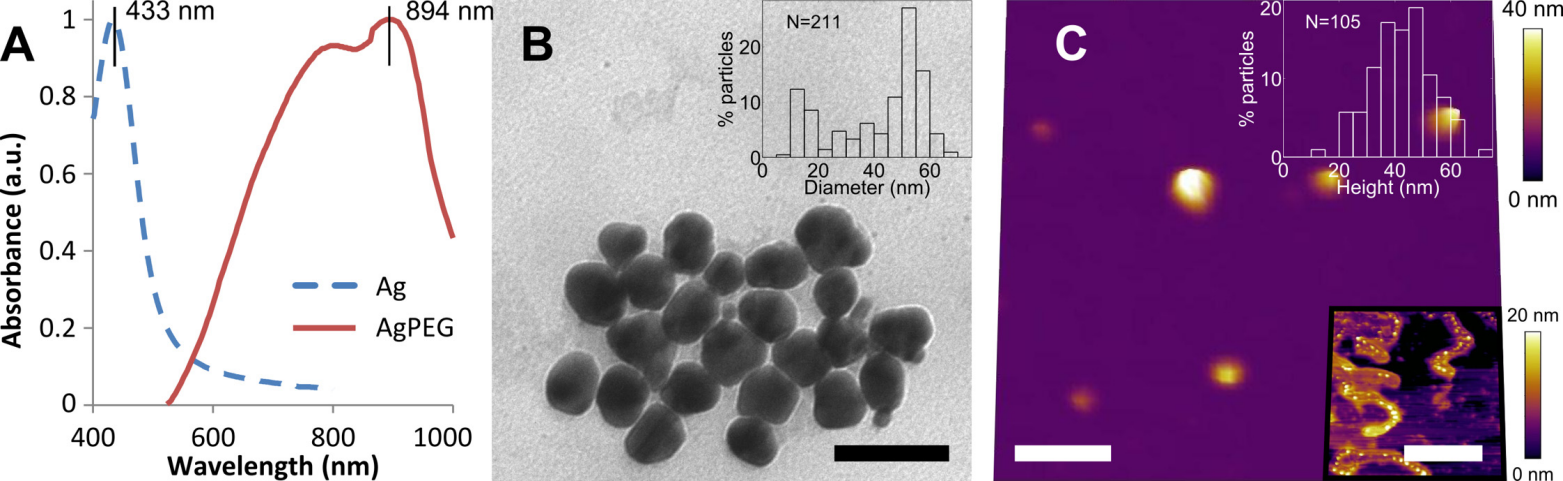
**Characterisation of AgPEG**. (**A**) UV-Vis absorbance spectrum showing a significantly shifted absorption peak at 894 nm. The original silver nanoparticle spectrum is shown for comparison. (**B**) TEM image showing homogeneous nanoparticles 100 days post-synthesis (scale bar 100 nm), with inset particle diameter distribution. (**C**) AFM topography image in air showing disperse homogeneous nanoparticles 100 days post-synthesis (scale bar 1 μm), with upper inset particle height distribution. Lower inset shows AgPEG nanoparticles dried in ethanol (scale bar 1 μm), revealing absorption of small nanoparticles in PEG.

Immediately after synthesis, we functionalised the nanoparticles with PEG via direct PEGylation, a common method to improve their stability [32]. The characterisation of the resulting AgPEG nanoparticles is shown in Figure 2. The UV-Vis spectrum shows a distinct redshift in the absorbance from 433 to 894 nm (Figure 2A). TEM characterisation gave a median metallic particle diameter of about 47 nm (Figure 2B). AFM characterisation gave a median particle height of about 40 nm (Figure 2C). The observed substantial absorbance shift has been reported during photo-induced conversion of nanospheres to nanoplates [16,37], although we found no evidence of nanoplates during both TEM and AFM characterisations. We speculate that this shift may be due to a complex coupled absorbance phenomenon due to the proximity of the nanoparticles linked with PEG. Drying the sample for AFM in the presence of ethanol, we indeed observed smaller seed particles absorbed by PEG surrounding the core silver nanoparticles (Figure 2C inset). Trapping seeds in this way will prevent further uncontrolled growth via ripening. The use of PEG as a functionalising agent is common, and its effect is attributed to steric repulsion between PEG chains adsorbed on nanoparticle surfaces [33,34]. We propose that it is the combination of steric repulsion and trapping of seeds that prevents further uncontrolled growth and improves stability. The larger particle size after functionalisation could be explained by the reported activity of PEG as a reducing agent of silver ions [33,38]. PEG may continue to catalyse ion reduction in the nanoparticle solution, consequently increasing nanoparticle size.

To explore the effects of non-steric stabilisations, we functionalised citrate-capped silver nanoparticles with gold to create bimetallic silver-gold nanoshells. Gold nanoparticles synthesised by the Turkevich method are known for their homogeneity [39], which suggests an increased stability between gold and citrate compared with silver and citrate.

### Silver-gold nanoshells: bimetallic stability I

**Figure 3:**
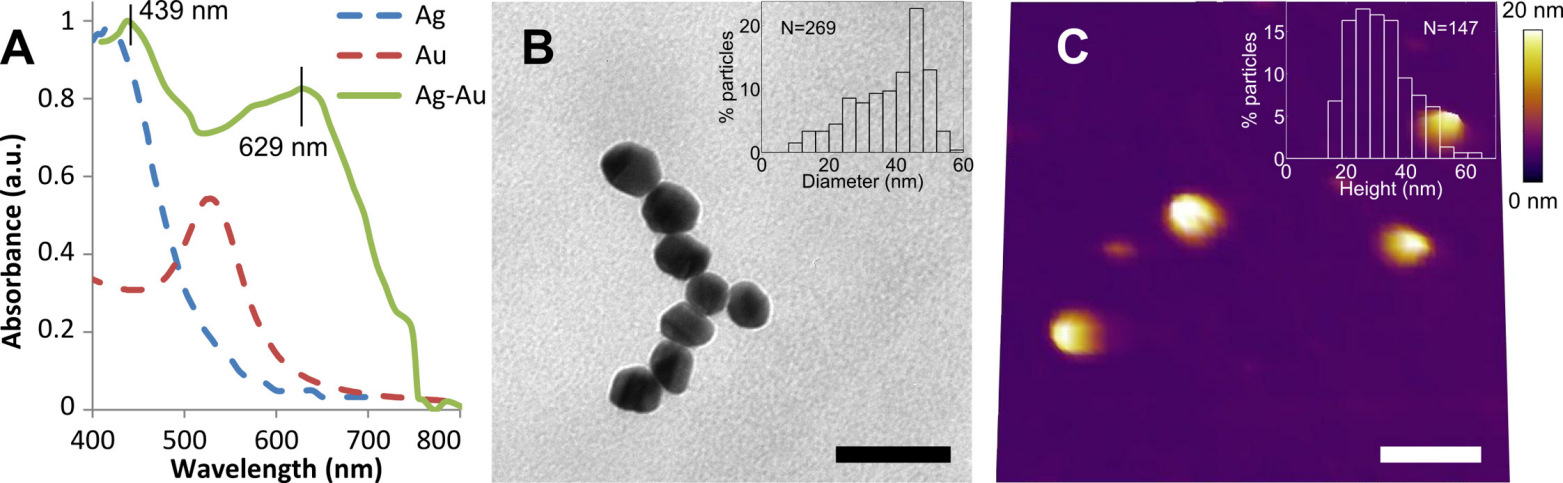
**Characterisation of bimetallic silver-gold nanoshells**. (**A**) UV-Vis absorbance spectra showing pure silver and pure gold nanoparticles for comparison and the Ag-Au bimetallic nanoparticle, with two absorption peaks at 439 and 629 nm. (**B**) TEM image showing homogeneous nanoparticles 100 days post-synthesis (scale bar 100 nm), with inset particle diameter distribution. (**C**) AFM topography image in air showing disperse homogeneous nanoparticles 100 days post-synthesis (scale bar 800 nm), with inset particle height distribution.

Two types of bimetallic nanoparticles can be obtained: (1) nanoparticles consisting of two metals with well-separated distributions called core-shell nanoparticles (nanoshells) and (2) nanoparticles with a homogeneous distribution of two metals called alloys [40]. Nanoshells can be obtained by successive reduction of two metals, where the first metal is reduced to form the core, and the second metal is reduced to form the shell [22]. Figure 3 shows the characterisation of the Ag-Au nanoshells obtained in this work. The UV-Vis spectrum shows two absorption peaks at 439 and 629 nm (Figure 3A). We observe a redshift in the gold peak from 526 to 629 nm and a smaller redshift in the silver peak from 410 to 439 nm. TEM and AFM data obtained 100 days post-synthesis gave a median particle diameter of about 39 nm (Figure 3B) and a median particle height of about 28 nm (Figure 3C), respectively. The small redshift in the silver absorbance peak is most likely due to an increased size of the core during the formation of the gold shell [17]. An increasing redshift in the gold absorption peak has been reported both experimentally and theoretically with an increasing gold shell thickness, although the chemical composition, dielectric environment and shape of the nanoshells will also affect the position of the absorbance peak [21,41-44]. Due to the sedimentation step during synthesis (see the 'Materials and methods' section), smaller seed-like particles were not observed. The obtained nanoshells were homogenous in both size and shape, in contrast to the pure silver nanoparticles. The higher valence of gold used (+3 compared with +1 for silver) may affect metal-citrate bonding, with more citrate ions able to bind to each metal ion at the surface, leading to increased stability.

To investigate whether a weak silver-citrate affinity at the nanoparticle surface is responsible for nanoparticle instability, we synthesised silver-chromium alloy nanoparticles through co-reduction, where chromium also has a higher valence (+3).

### Silver-chromium alloy nanoparticles: bimetallic stability II

**Figure 4:**
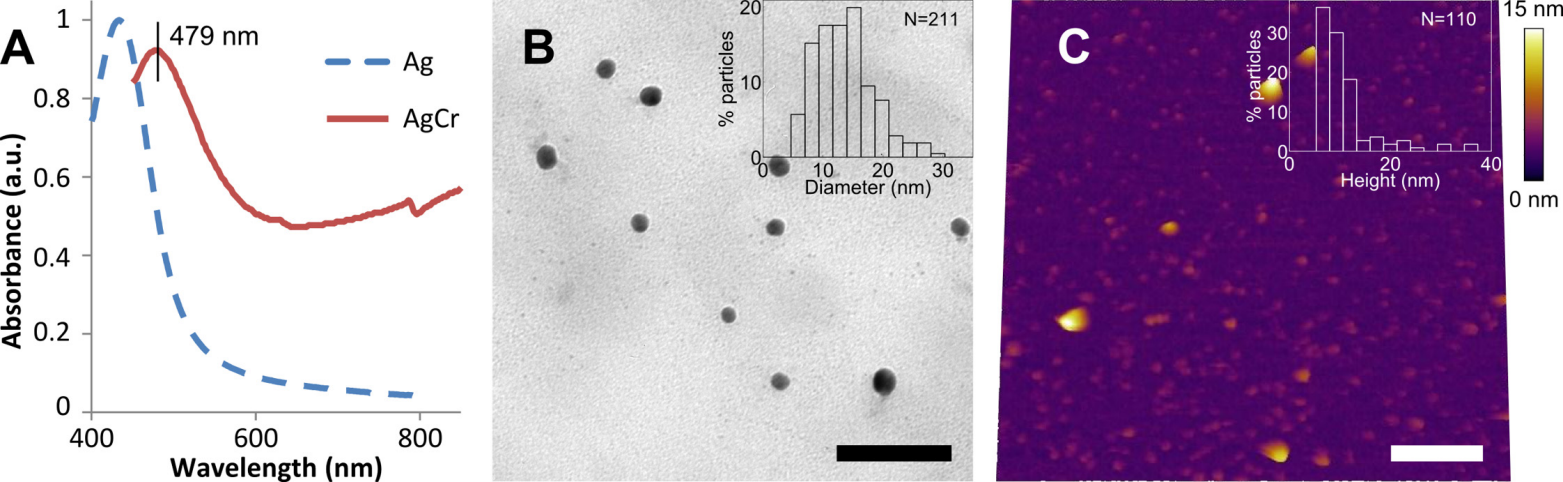
**Characterisation of bimetallic silver-chromium alloy nanoparticles**. (**A**) UV-Vis absorbance spectrum showing an absorption peak at 479 nm. The spectrum for pure silver nanoparticles is shown for comparison. (**B**) TEM image showing disperse nanoparticles 100 days post-synthesis (scale bar 100 nm), with inset particle diameter distribution. (**C**) AFM topography image in liquid showing disperse nanoparticles 100 days post-synthesis (scale bar 150 nm), with inset particle height distribution.

The characterisation of obtained AgCr alloy nanoparticles is shown in Figure 4. The UV-Vis spectrum shows a redshift in the plasmon resonance absorption peak from 433 to 479 nm and a broadening of the spectrum (Figure 4A). TEM and AFM characterisations 100 days post-synthesis gave a median particle diameter of about 12 nm (Figure 4B) and a median particle height of about 7 nm (Figure 4C), respectively. The broadening of the plasmon absorption peak corresponds to the low but broad absorption of Cr^3+^, and the redshift in the peak is likely due to a combination of the altered composition and dielectric function of the alloy [44,45]. The diameter and height distributions were well confined below 40 nm; however, smaller seeds were numerous. Despite the presence of these seeds, the alloy nanoparticles remained stable in size, shape and dispersibility confirming that the presence of a higher valence metal (chromium) on the nanoparticle surface significantly improves stability, without requiring the complete capping of the core metal.

### Improved stability in shapes and sizes of nanoparticles

**Figure 5:**
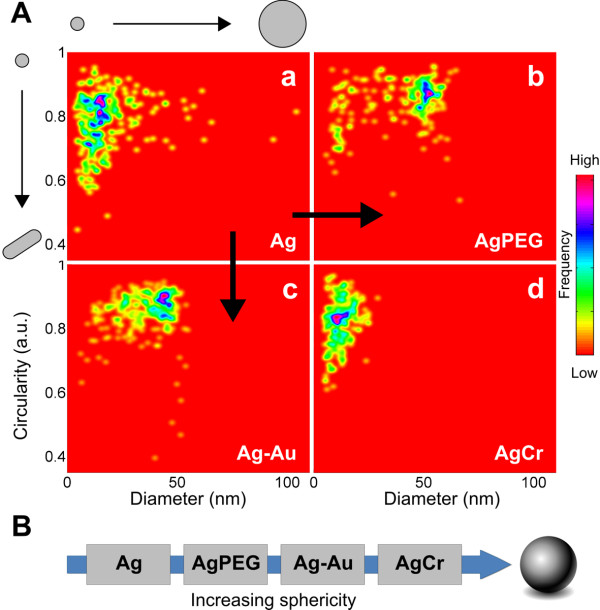
**Comparative study of particle size and shape**. (**A**) 2-D histogram of nanoparticle diameter correlated to 2-D circularity from TEM data. A circularity of 1 indicates a spherical particle, and 0.4 indicates a rod-shaped particle. More dense regions appear purple and represent larger populations. (a) Ag, (b) AgPEG, (c) Ag-Au and (d) AgCr nanoparticles. (**B**) Results of Kruskal-Wallis hypothesis testing on 3-D particle sphericity from combined AFM and TEM data.

To quantify the effects previously described on the size and shape of the obtained nanoparticles, we analysed the TEM data before combining and analysing TEM and AFM data. First, we analysed the TEM nanoparticle profiles. Figure 5 shows two-dimensional (2-D) histograms of nanoparticle diameters and circularities calculated for the nanoparticles that are synthesised in this study. In a TEM image, circularity is defined as the ratio of the area of a nanoparticle with its perimeter, determined via segmentation (see the 'Materials and methods' section). Circularity ranges from a value of 1 for a circular object (spherical or disc-shaped nanoparticle, with the latter eliminated due to the absence of observed discs) to 0 for a highly elongated object (rod-shaped nanoparticle). In our work, we did not observe any single nanoparticle with a circularity lower than 0.4. Silver nanoparticles ((a) of Figure 5A) were the most heterogenic in size and shape. Functionalising silver nanoparticles with PEG ((b) of Figure 5A) resulted in particles with an increased diameter and circularity, which are both more narrowly distributed, indicating the predominance of a single species of nanoparticle. AgPEG nanoparticles around 15 nm in diameter observed in the histogram probably remain unfunctionalised. Capping with gold to create the Ag-Au nanoshells ((c) of Figure 5A) resulted in particles with an increased and more narrowly distributed diameter and circularity, again indicating the tendency towards a single species. A small presence of rods was observed, which was expected in the gold functionalisation step as by-products of pure gold rod formation. Finally, AgCr alloy nanoparticles ((d) of Figure 5A) were the most narrowly distributed in size, with no particle diameters above 40 nm. We predominantly observed circular objects although the circularity metric is compromised as particle diameters become smaller (see the 'Materials and methods' section for more details); this explains the broad range of obtained circularities in the case of the smaller AgCr nanoparticles.

Second, we combined TEM diameters with AFM heights to ascertain particle sphericities in three dimensions, where a higher particle sphericity indicates a more stable, homogenous growth process. We found (Figure 5B, with details in Additional file 1) that the nanoparticle sphericities increased with each stabilisation strategy, i.e. unfunctionalised nanoparticles were the least stable, followed by PEG-functionalised, silver-gold nanoshells and silver-chromium alloy nanoparticles.

## Conclusions

Due to the highly reactive nature of silver ions and the multiple roles that citrate plays during synthesis, it has been difficult to control the size and shape of citrate-reduced silver nanoparticles without further functionalisation. In this work, we synthesise a 15 nm quasi-spherical silver nanoparticle directly in water without organic solvents. After 100 days in solution, the nanoparticles exhibited increased heterogeneity due to their inherent instability. We used PEG as an organic capping agent and gold as an inorganic capping agent. In both cases, we are effectively able to control further growth and stabilisation of the nanoparticles. We found that the addition of PEG not only stabilises the nanoparticles by steric repulsion and trapping of seeds, but also allows controlled further growth of nanoparticles, improving homogeneity in nanoparticle size, shape and stability. In the case of Ag-Au nanoshells, the higher electron affinity of gold may lead to a stronger gold-citrate interaction at the nanoparticle surface compared with that in the citrate-capped silver nanoparticles, consequently improving size and shape homogeneity and long-term stability. Similarly to gold, the higher valence of chromium may lead to a stronger chromium-citrate interaction at the nanoparticle surface. The alloying process enables the production of stable silver-based nanoparticles without the need for additional functionalisation/capping.

The findings of this work enable further fundamental research on the effects of material, size and shape on nanoparticle behaviour. The ability to understand and control the nanoparticle/liquid interface allows the possibility of studying the role of nanoparticles in further applications where electrolytes are an important consideration for e.g. water treatment and biomedical use (due to the presence of salts in physiological solutions), which is particularly timely for quantitative toxicity studies, given the current and future widespread production and application of silver nanostructures.

## Competing interests

The authors declare that they have no competing interests.

## Authors' contributions

DBB, AIC and ST carried out nanoparticle synthesis, functionalisation and UV-Vis characterisation. APZS carried out the AFM characterisation and AFM/TEM analysis of nanoparticles. SAC helped with AFM characterisation and conceptual discussions. SC constructed and performed the statistical analysis of nanoparticle sphericity. APZS and ST co-wrote the manuscript. ST conceived the study. All authors read and approved the final manuscript.

## Supplementary Material

Additional file 1 **Supplementary data**. Bootstrap statistical analysis of AFM/TEM data, including non-parametric Kruskal-Wallis hypothesis testing. Click here for file
