# Supplementary material for: Three strategies to stabilise nearly monodispersed silver nanoparticles in aqueous solution
Source: Nanoscale Res Lett. 2012 Feb 22;7(1):151. doi: 10.1186/1556-276X-7-151 (PMC3351018; doi:10.1186/1556-276X-7-151)
Supplement: Additional file 1 — Supplementary data. Bootstrap statistical analysis of AFM/TEM data, including non-parametric Kruskal-Wallis hypothesis testing. [file 1556-276X-7-151-S1.PDF]

Three strategies to stabilise nearly monodispersed silver nanoparticles in aqueous solution

Amadeus PZ Stevenson[1], Duani Blanco Bea[2], Sergi Civit[3], Sonia Antoranz Contera[1,4], Alberto Iglesias Cerveto[2] and Sonia Trigueros*[1,4]

[1]Department of Physics, University of Oxford, Parks Road, Oxford, OX1 3PU, UK

[2]Department of Materials, National Centre for Scientific Research, PO Box 6414, Avenida 25 and 158, Cubanacán, Playa, Havana, CP 12100, Cuba

[3]Department of Statistics, University of Barcelona, Avenida Diagonal 645, Barcelona, 08028, Spain

[4]Institute of Nanoscience for Medicine, Oxford Martin School, 34 Broad Street, University of Oxford, Oxford, OX1 3BD, UK

*Corresponding author: s.trigueros1@physics.ox.ac.uk

Supplementary Data

Bootstrapping is a very useful tool in statistics in cases when there is doubt that distributional assumptions and asymptotic results are valid and accurate [S1]. The fundamental concept in bootstrapping is in building a sampling distribution for a particular statistic by resampling from the data at hand. Based on the two independent samples obtained by TEM and AFM we modelled nanoparticle sphericity by constructing an ellipse with diameter from TEM and height from AFM, and calculate the corresponding statistic eccentricity (ε, eq (1)):

$$\varepsilon = \sqrt{\frac{a^2 - b^2}{a^2}} = \sqrt{1 - \left(\frac{b}{a}\right)^2} \quad o < \varepsilon < 1 \qquad (1)$$

where a and b are an ellipse's half major and minor axes (diameter and height) respectively.

The eccentricity of an ellipse is a measure of how circular the ellipse is. As the eccentricity value ranges from 0 to 1, the ellipse ranges from circular to highly elongated. According to eq (1) and non-parametric bootstrap techniques (B = 1000 resampling) we plot the distribution of our bootstrapped

statistic as a histogram (Fig. S1A) and we summarise the results by using the percentile bootstrap confidence intervals (95% confidence level) (Table S1). In addition, we address the question of interest "How spherical are the nanoparticles synthesised in this study?" according to the the Kruskal-Wallis hypothesis test. The Kruskal-Wallis test is the non-parametric analog to One-way Analysis of Variance. This test is used to determine if there are "significant" differences among the population medians (instead of the population means). The Kruskal-Wallis H statistic is an overall test statistic that enables one to test the general hypothesis that all population medians are equal. However, the investigator is not extremely interested in this general hypothesis but is interested in comparisons amongst the individual groups. According to that we compare the nanoparticle types to establish a ranking of eccentricity, and hence sphericity (where a low eccentricity corresponds to a high sphericity). We find (Fig. S1B, Table S2) that the silver-chromium alloy nanoparticles are the most spherical, followed by the silver-gold nanoshells, PEG-functionalised silver and finally pure silver nanoparticles.

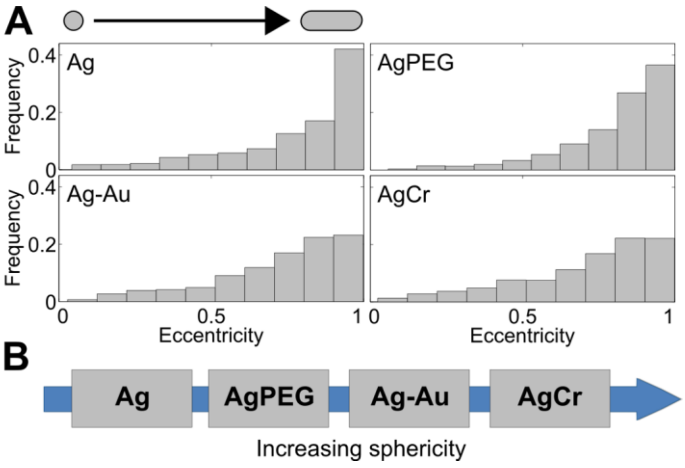

Fig. S1. Statistical analysis of nanoparticle shape from combining TEM (diameter) and AFM (height) data distributions. (A) Eccentricity bootstrap distributions obtained for each nanoparticle type. High frequencies to the left x-axes (low eccentricity values) indicate a spherical nanoparticle. (B) Results of non-parametric Kruskal-Wallis hypothesis testing on particle eccentricity (a low eccentricity corresponds to a high sphericity).

Table S1

Bootstrap eccentricity percentile confidence intervals (95% confidence level).

Nanoparticle	Percentile CI (95%)
Ag	[0.25, 0.99]
AgPEG	[0.30, 0.99]
Ag-Au	[0.21, 0.98]

AgCr	[0.17, 0.98]

Table S2

Kruskal Wallis hypothesis test and Multiple comparisons (significance level= 0.05).

Eccentricity (e) test:

Kruskal-Wallis: Multiple Comparisons

Kruskal-Wallis Test on the data

Group	N	Median	Ave Rank	Z
AgCr	1000	0.7741	1706.8	-9.29
Ag	1000	0.8584	2249.0	7.86
AgPEG	1000	0.8522	2263.4	8.31
AgAu	1000	0.7805	1782.9	-6.88
Overall	4000		2000.5	

H = 198.31 DF = 3 **P = 0.000**

H = 198.31 DF = 3 P = 0.000 (adjusted for ties)

Kruskal-Wallis: All Pairwise Comparisons

Comparisons:	6
Ties:	73
Family Alpha:	0.2
Bonferroni Individual Alpha:	0.033
Bonferroni Z-value (2-sided):	2.128

Sign confidence interval for median

			Achieved	Confidence Interval		
	N	Median	Confidence	Lower	Upper	Position
AgCr	1000	0.7741	0.8628	0.7551	0.7830	477
			0.8676	0.7551	0.7832	NLI
			0.8787	0.7550	0.7838	476
Ag	1000	0.8584	0.8628	0.8479	0.8689	477
			0.8676	0.8478	0.8691	NLI
			0.8787	0.8476	0.8695	476
AgPEG	1000	0.8522	0.8628	0.8451	0.8638	477
			0.8676	0.8451	0.8639	NLI

			0.8787	0.8451	0.8640	476
AgAu	1000	0.7805	0.8628	0.7694	0.7931	477
			0.8676	0.7693	0.7934	NLI
			0.8787	0.7690	0.7941	476

Kruskal-Wallis: Conclusions

The following groups showed significant differences (adjusted for ties):

Groups	Z vs. Critical value	P-value
AgCr vs. AgPEG	10.7775 >= 2.128	0.000
AgCr vs. Ag	10.4985 >= 2.128	0.000
AgPEG vs. AgAu	9.3035 >= 2.128	0.000
Ag vs. AgAu	9.0245 >= 2.128	0.000

The graph displays boxplots of the groups with their sign confidence intervals for the medians. This graph is extremely useful because you can visually see the **eccentricity values** of the nanoparticles with respect to the others **(a low eccentricity corresponds to a high sphericity)**

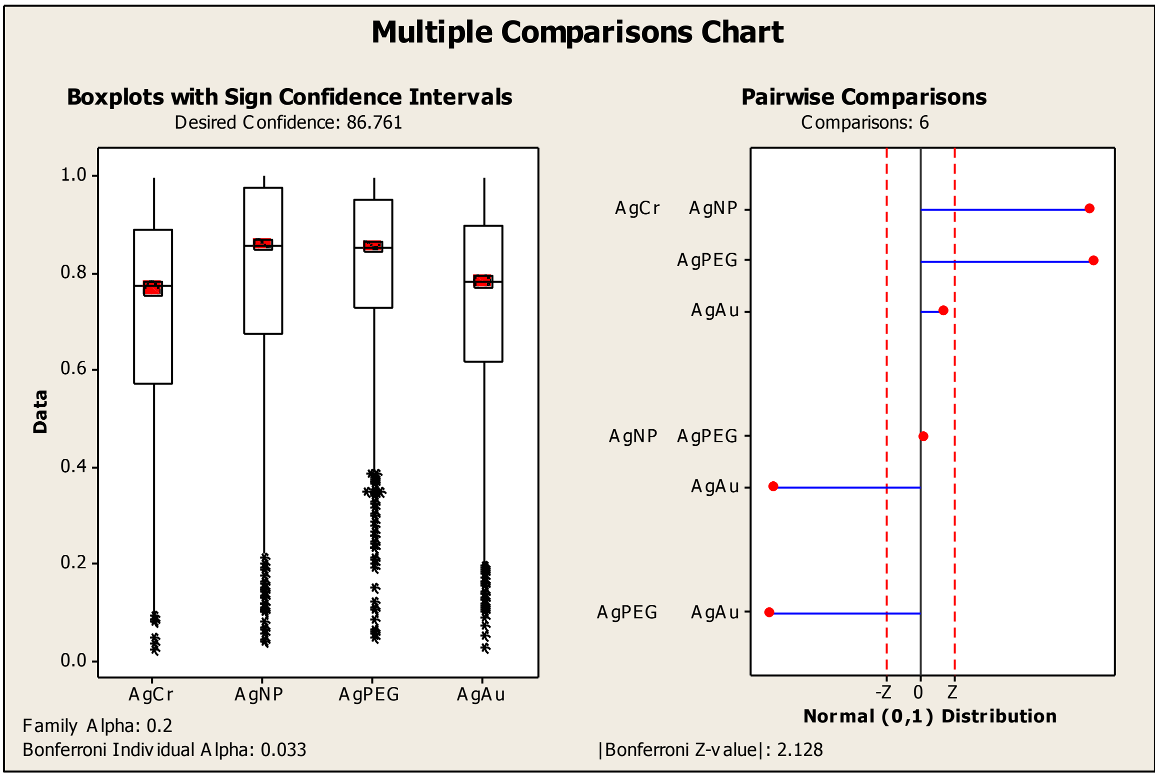

Due to the presence of extreme values in our dataset we performed bootstrapping by weighting frequency values. In other words, once the value becomes extreme enough, the weight for that observation becomes very small in the resampling procedure so the influence of the observation is minimal. These weights are the relative frequencies of particle diameters from TEM and particle heights from AFM.

References

S1. Efron B, Tibshirani RJ: *An introduction to the Bootstrap.* London: Chapmann & Hall; 1993.
